# Situational and Positional Effects on the Technical Variation of Players in the UEFA Champions League

**DOI:** 10.3389/fpsyg.2020.01201

**Published:** 2020-06-19

**Authors:** Qing Yi, Miguel-Ángel Gómez, Hongyou Liu, Binghong Gao, Fabian Wunderlich, Daniel Memmert

**Affiliations:** ^1^School of Physical Education and Sport Training, Shanghai University of Sport, Shanghai, China; ^2^Shanghai Key Lab of Human Performance, Shanghai University of Sport, Shanghai, China; ^3^Facultad de Ciencias de la Actividad Física y del Deporte, Universidad Politécnica de Madrid, Madrid, Spain; ^4^Institute of Training and Computer Science in Sport, German Sport University Cologne, Cologne, Germany; ^5^School of Physical Education and Sports Science, South China Normal University, Guangzhou, China

**Keywords:** football, soccer, playing position, situational variable, match analysis

## Abstract

This study aimed to identify the situational and positional effects on the variation of players’ technical performance in the UEFA Champions League from a long-term perspective. The technical performance of full match observations from outfield players in the UEFA Champions League from season 2009/2010 to 2016/2017 was analysed. The coefficient of variation of each variable of each player in each season was calculated to evaluate the match-to-match variation of technical performance. The variation of technical performance between players was compared across five playing positions and five situational variables using the non-clinical magnitude-based inference. Results showed that variables related to goal scoring, passing and organising from five playing positions showed a relatively higher variation among five competing contexts (ES: −0.72 ± 0.38 – 0.82 ± 0.61). Quality of team, quality of opponent and match outcome showed relatively greater influences than competition stage and match location on the variation of a player’s technical performance (ES: −0.72 ± 0.38 – 0.57 ± 0.56). The technical performances of wide players (full backs and wide midfielders) were more variable between the group and knockout stage (ES: −0.37 ± 0.32 – 0.28 ± 0.19). This study provides an important understanding of the associations among the variation of technical indicators, playing positions and situational variables. These profiles of technical variation could be used by coaches and analysts for talent identification, player recruitment, pre-match preparation and post-match evaluation.

## Introduction

Performance analysis in sports is a powerful communication and feedback tool to prepare or guide players during practice (Memmert and Rein, 2018). The investigation of technical parameters provides an objective understanding of actual match performance and can help to explain the differences between successful and unsuccessful match performances (McGarry et al., 2013; Memmert et al., 2017).

It is well known that match actions or events within individual players are characterised by high variability over successive matches, and the variation can be a result of internal and external factors (Kempton et al., 2015; Memmert and Raabe, 2018). Variability analysis of performance indicators is an effective way to measure the stability and consistency of a player (Bush et al., 2015b). The smaller the within-player variation, the easier to identify the change of his/her match performance (Hopkins, 2000). The within-subject variation is best represented using the coefficient of variation (CV) (Hopkins, 2000), as was previously accounted for quantifying the performance variability of players or teams (Rampinini et al., 2007; Bush et al., 2015b; Liu et al., 2016). This approach allows one to identify relationships between the variation of performance indicators and the match performance of players or teams, and key performance indicators could be identified.

The competing situations have been considered as the external factors that may cause within-subject variation in the performance of players/teams, such as match location, quality of the team and quality of the opponent (Jones et al., 2004; Lago and Martin, 2007). Players need to adapt psychologically and physiologically to these competing scenarios (Eccles et al., 2009), and their variation of technical and physical performance can be influenced by these situational conditions at a behavioural level (Gómez et al., 2013; Liu et al., 2015). In addition, the differences of players’ performance can also be found in different match outcomes, i.e., win, draw and defeat (Castellano et al., 2012; Gómez et al., 2012). Meanwhile, competition stage is another situational variable that has great importance on players’ performance, as teams’ opponents in the knockout stage are generally stronger than those in the group stage (Gómez et al., 2013). A prior study has identified the difference in technical performance between regular season and playoffs in basketball (Sampaio and Janeira, 2003). To the best of our knowledge, previous studies paid little attention to the differences in variability of the performance of football players in different competition stages (e.g., group stage/knockout stage).

Moreover, the assessment of players’ match performances has to be seen in view of the player’s position on the pitch. The team’s success is the result of intelligent tactics combined with an appropriate level of technical and physical performance of players from different playing positions playing different roles in a football match (Bush et al., 2015a; Yi et al., 2018). Therefore, positional differences should not be disregarded; otherwise, a wealth of valuable information will be hidden and restrict the understanding of the players’ performance (Low et al., 2019). In particular, Sarmento et al. (2014) reviewed prior studies and concluded that players’ positions were previously classified into either three (defenders, midfielders and forwards) or five groups (central defenders, external defenders, central midfielders, external midfielders and forwards). The latter category may provide more detailed information about the players’ match performances on the pitch as players are given a more specific tactical role in a modern football match (Yi et al., 2018).

Studies about the differences in technical variation either considering situational variables or playing positions are well documented (Di Salvo et al., 2007; Bush et al., 2015a; Liu et al., 2016). Nevertheless, further research is necessary to assess the interactive effects among playing positions and situational variables on players’ technical variation and to inform how the effects of these two factors can be interpreted in a more detailed way (Sarmento et al., 2014). Additionally, the previous systematic review of available literature confirmed the importance of situation variables and playing position factors from players’ physical performances. However, relatively less attention was obtained for the technical performance factors (Sarmento et al., 2014).

Therefore, the current study aimed to identify the differences of players’ technical variation in the UEFA Champions League from a long-term perspective (eight seasons) across five players’ specific field positions and five competing situations. This comprehensive analysis based on a large dataset can provide important insights into the highly dynamic and complex nature of a football match, assisting coaches and analysts to better understand the influences of situational variables and playing positions during the evaluation of players’ technical performance.

## Materials and Methods

### Data Resource

This study involved the analysis of publicly available data obtained from a football statistics website “whoscored.com^1^”. Match statistics included in this website were provided by the OPTA Sports Company (London, United Kingdom), and the consistency of match statistics between these two could be assured. The tracking system (OPTA Client System) has been tested to have acceptable reliability in coding players’ match actions and events (Liu et al., 2013). The current study was performed in accordance with the Declaration of Helsinki and maintains the anonymity of the players according to European data protection law. The ethical approval for this study was obtained from the ethics committee of the Shanghai University of Sport (11DZ2261100).

### Sample and Variables

#### Sample and Characteristics

Technical performance data of players from 1,000 matches (group stage: 768 matches; knockout stage: 232 matches) in the UEFA Champions League from season 2009/2010 to 2016/2017 were collected. Considering the positional specificities for goalkeepers, the goalkeepers’ data were excluded from the database. Only the outfield players who played at least two full matches in the group stage or knockout stage at each season were included, which finally limited the subjects to 3,276 players (*n* = 2,389 players from the group stage, *n* = 887 players from the knockout stage) across 12,908 full match observations (*n* = 10,122 from the group stage, *n* = 2,786 from the knockout stage). Due to the limitation of the number of matches in the knockout stage, players’ match data from the knockout stage were only included in the comparison between competition stages. For the analysis of the other four situational variables, only the match data of players from the group stage were included.

#### Technical Variables

Twenty-five technical performance-related variables were studied and were divided into four groups based on the available related literature (Lago-Peñas et al., 2010; Castellano et al., 2012; Yi et al., 2019a,b,c). The grouping information see Table 1.

**TABLE 1:** The classification of technical variables.

Categories	Variables
Goal scoring	Shot, shot on target
Attacking	Dispossessed, unsuccessful touches, fouled, aerial won, dribble, offside
Passing and organising	Assist, touch, key pass, pass accuracy (%), pass, cross, accurate cross, long ball, accurate long ball, through ball, accurate through ball
Defending	Yellow card, total tackle, interception, clearance, blocked shot, foul.

#### Situational Variables

Five situational variables were included: (1) competition stage (group stage/knockout stage); (2) match location (home/away); (3) quality of team (qualified team/non-qualified team); (4) quality of opponent (qualified opponent/non-qualified opponent); and (5) match outcome (win/draw/defeat). Thirty-two teams that compete in the group stage were drawn into eight groups of four each season; those two teams that qualified for the knockout stage were considered as qualified teams, whereas the other two teams were considered as non-qualified teams.

#### Playing Positions

Playing positions were categorised as central defender (group stage: *N*_1_ = 865 players, *n*_1_ = 2,667 full match observations, knockout stage: *N*_2_ = 286 players, *n*_2_ = 727 observations), full back (*N*_1_ = 787, *n*_1_ = 2,505, *N*_2_ = 228, *n*_2_ = 663), wide midfielder (*N*_1_ = 550, *n*_1_ = 1,053, *N*_2_ = 155, *n*_2_ = 291), central midfielder (*N*_1_ = 1,040, *n*_1_ = 2,794, *N*_2_ = 322, *n*_2_ = 774), and forward (*N*_1_ = 504, *n*_1_ = 1,103, *N*_2_ = 133, *n*_2_ = 331). The classification of positions was based on the available research (Liu et al., 2016; Yi et al., 2018).

### Statistical Analysis

The CV of each variable of each player in the group stage or knockout stage of each season was calculated to present the variation of match performance within a player during the past eight seasons (Hopkins, 2000; Bush et al., 2015b; Kempton et al., 2015). If the mean of a count value of an action or event of a player is 0 (e.g., 0 shot in two matches), the CV of this action or event of this player was expressed as a missing value (Liu et al., 2016). The non-clinical magnitude-based inferences were used to identify the differences in players’ technical variation across five playing positions and five competing situations. Significant differences were evaluated using the standardised smallest worthwhile change, which was calculated by 0.2 times the between-subject standard deviation. Comparisons between groups were conducted using the spreadsheet developed by Hopkins (Hopkins, 2007; Hopkins et al., 2009), and the estimated magnitudes of difference in means and their 90% confidence limits were presented in standardised units and were evaluated qualitatively with the following scale: trivial, <0.2; small, 0.2–0.6; moderate, 0.6–1.2; large, 1.2–2.0; and very large, >2.0 (Batterham and Hopkins, 2006). The likelihood of the effect to be clear was defined as follows: 25%–75%, possibly; 75%–95%, likely; 95%–99.5%, very likely; and 99.5%–100%, most likely (Hopkins et al., 2009).

## Results

**FIGURE 1:**
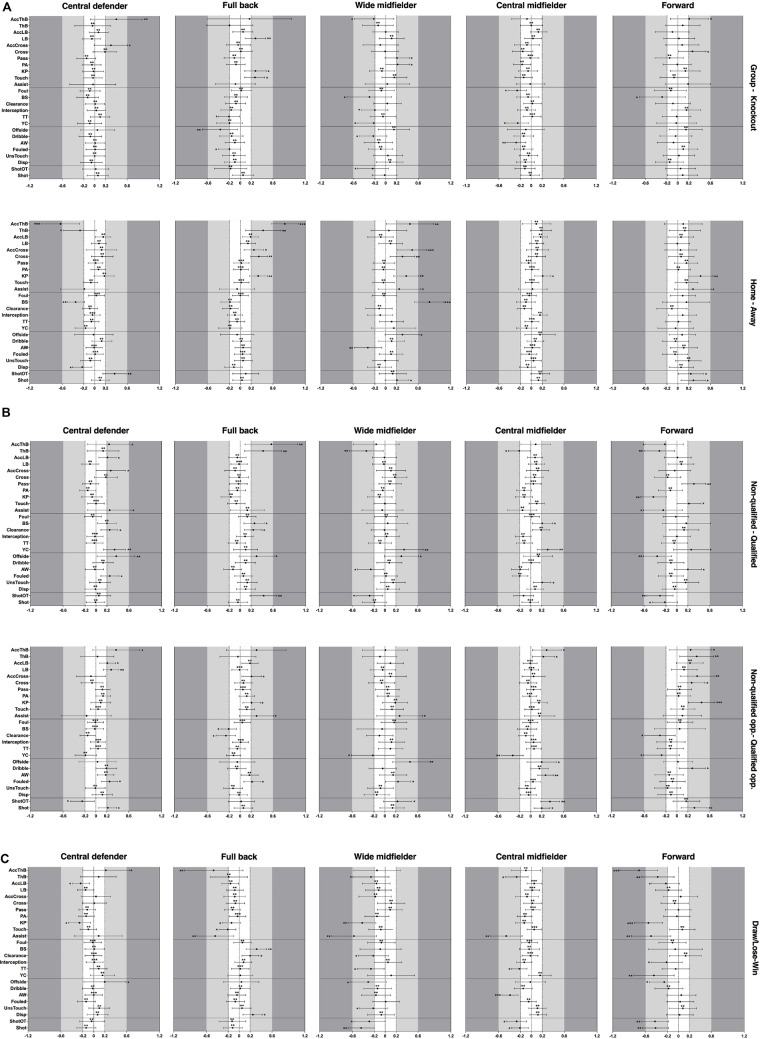
Standardised effects of playing positions and competing situations on the technical variation (CV) of players. Part labels **(A–C)** displayed the differences in technical variation of 5 playing positions under the competing situations of competition stage and match location, quality of team and opponent, match outcome, respectively. Bars are 90% confidence intervals. Asterisks indicate the likelihood for the magnitude of the true differences in mean as follows: *possible; **likely; ***very likely; ****most likely. Asterisks located in the trivial area denote for trivial differences. ShotOT, shot on target; Disp, player is dispossessed on the ball by an opponent-no dribble involved; UnsTouch, bad control; AW, aerial won; YC, yellow card; TT, total tackle; BS, blocked shot; KP, key pass; PA, pass accuracy in %; AccCross, accurate cross pass; LB, long ball; AccLB, accurate long ball; ThB, through ball; AccThB, accurate through ball.

Figure 1 shows a graphical representation of the differences in technical variation between players from five playing positions under five competing situations. Table 2 gives a summary of those technical variables that revealed significant differences. The full set of descriptive statistics of the players’ technical variation can be found in the Supplementary Material.

**TABLE 2:** Substantial differences of players’ match performance variation across five playing positions and five competing situations.

Position	Group – Knockout			Home – Away			Non-qualified – Qualified			Non-qualified Opp. – Qualified Opp.			Draw/Lose – Win		
	Variable	Effect size	Inference	Variable	Effect size	Inference	Variable	Effect size	Inference	Variable	Effect size	Inference	Variable	Effect size	Inference
CD	AccThB	0.40 ± 0.49	S**	AccThB	−0.63 ± 0.35	M***	YC	0.34 ± 0.20	S**	LB	0.28 ± 0.14	S**	Offside	0.20 ± 0.38	S*
	AccCross	0.30 ± 0.31	S*	BS	−0.35 ± 0.16	S**	Offside	0.37 ± 0.34	S**	Shot	0.22 ± 0.16	S*	KP	−0.26 ± 0.20	S*
				ShotOT	0.37 ± 0.22	S**	Assist	0.25 ± 0.40	S*	ShotOT	−0.25 ± 0.22	S*	AccLB	−0.24 ± 0.16	S*
				Disp	−0.23 ± 0.18	S*	Clearance	0.25 ± 0.16	S*	Fouled	0.25 ± 0.15	S*	AccThB	0.22 ± 0.42	S*
				ThB	−0.27 ± 0.31	S*	Fouled	0.25 ± 0.16	S*	AccLB	0.21 ± 0.14	S*			
							AccCross	0.27 ± 0.27	S*	AccThB	0.37 ± 0.43	S*			
							AccLB	0.21 ± 0.16	S*						
							AccThB	0.24 ± 0.38	S*						
FB	LB	0.28 ± 0.19	S**	AccThB	0.82 ± 0.61	M***	ShotOT	0.43 ± 0.25	S**	Assist	0.30 ± 0.31	S*	Assist	−0.44 ± 0.34	S**
	Offside	−0.37 ± 0.32	S**	KP	0.33 ± 0.16	S**	ThB	0.42 ± 0.34	S**	BS	−0.21 ± 0.14	S*	BS	0.32 ± 0.19	S**
	TT	−0.21 ± 0.19	S*	Cross	0.34 ± 0.16	S**	AccThB	0.57 ± 0.56	S**	Fouled	0.22 ± 0.15	S*	AccThB	−0.47 ± 0.54	S**
	Fouled	−0.20 ± 0.20	S*	ThB	0.42 ± 0.33	S**	Clearance	0.24 ± 0.16	S*	KP	0.21 ± 0.16	S*	Disp	0.25 ± 0.18	S*
	Touch	0.27 ± 0.18	S*	AccCross	0.25 ± 0.18	S*	BS	0.26 ± 0.18	S*	AccCross	0.21 ± 0.18	S*	Clearance	0.20 ± 0.15	S*
	KP	0.28 ± 0.20	S*				Offside	0.30 ± 0.32	S*	Clearance	−0.26 ± 0.18	S*	Touch	−0.21 ± 0.15	S*
WM	YC	−0.22 ± 0.31	S*	BS	0.81 ± 0.39	M***	YC	0.35 ± 0.35	S**	Offside	0.46 ± 0.33	S**	Assist	−0.57 ± 0.41	S**
	ShotOT	−0.24 ± 0.28	S*	AccCross	0.49 ± 0.27	S***	ThB	−0.34 ± 0.31	S**	Assist	0.27 ± 0.42	S*	KP	−0.42 ± 0.26	S**
	Interception	−0.20 ± 0.24	S*	KP	0.38 ± 0.23	S**	ShotOT	−0.28 ± 0.24	S*	YC	−0.22 ± 0.39	S*	Shot	−0.44 ± 0.24	S**
	BS	−0.30 ± 0.42	S*	Cross	0.31 ± 0.23	S**	AW	−0.26 ± 0.24	S*	ShotOT	0.23 ± 0.26	S*	ShotOT	−0.29 ± 0.28	S*
	Dribble	−0.23 ± 0.25	S*	AccThB	0.45 ± 0.44	S**	Offside	0.30 ± 0.31	S*	Fouled	0.24 ± 0.23	S*	UnsTouch	−0.22 ± 0.25	S*
	PA	0.22 ± 0.22	S*	AW	−0.33 ± 0.25	S**							TT	−0.26 ± 0.25	S*
	Pass	0.21 ± 0.22	S*	Assist	0.25 ± 0.40	S*							Clearance	−0.22 ± 0.28	S*
	AccThB	−0.22 ± 0.37	S*	Shot	0.21 ± 0.22	S*							Offside	−0.31 ± 0.33	S*
				Offside	0.31 ± 0.32	S*							ThB	−0.26 ± 0.33	S*
CM	AW	−0.28 ± 0.17	S**	KP	0.21 ± 0.15	S*	YC	0.31 ± 0.19	S**	YC	−0.33 ± 0.19	S**	AW	−0.39 ± 0.15	S***
	YC	−0.25 ± 0.20	S*				UnsTouch	0.21 ± 0.16	S*	ShotOT	0.35 ± 0.18	S**	Assist	−0.46 ± 0.29	S**
	Foul	−0.26 ± 0.17	S*				BS	0.21 ± 0.18	S*	AW	0.27 ± 0.15	S**	Shot	−0.21 ± 0.15	S*
							ThB	−0.21 ± 0.20	S*	Shot	0.20 ± 0.14	S*	ShotOT	−0.27 ± 0.18	S*
										Offside	0.20 ± 0.27	S*	TT	−0.22 ± 0.14	S*
										ThB	0.23 ± 0.20	S*	ThB	−0.27 ± 0.21	S*
										AccThB	0.29 ± 0.26	S*			
FW	BS	−0.29 ± 0.48	S*	KP	0.42 ± 0.23	S**	ShotOT	−0.31 ± 0.24	S**	KP	0.45 ± 0.24	S***	AccThB	−0.72 ± 0.38	M***
	Cross	0.27 ± 0.25	S*	Assist	0.28 ± 0.34	S*	Offside	−0.36 ± 0.25	S**	Shot	0.32 ± 0.23	S**	KP	−0.55 ± 0.25	S***
	AccThB	0.21 ± 0.36	S*	Shot	0.29 ± 0.23	S*	KP	−0.43 ± 0.24	S**	AccCross	0.37 ± 0.31	S**	Assist	−0.50 ± 0.36	S**
				ShotOT	0.24 ± 0.23	S*	Pass	0.31 ± 0.22	S**	ThB	0.36 ± 0.31	S**	YC	−0.45 ± 0.37	S**
							ThB	−0.32 ± 0.28	S**	YC	−0.28 ± 0.33	S*	Shot	−0.42 ± 0.24	S**
							Assist	−0.25 ± 0.36	S*	Clearance	−0.31 ± 0.28	S*	ShotOT	−0.43 ± 0.25	S**
							YC	0.26 ± 0.31	S*	Dribble	0.28 ± 0.24	S*	ThB	−0.38 ± 0.30	S**
							Shot	−0.22 ± 0.23	S*	Cross	0.27 ± 0.24	S*	Interception	−0.22 ± 0.29	S*
							AW	0.20 ± 0.25	S*	AccLB	0.24 ± 0.25	S*	Offside	−0.26 ± 0.27	S*
							Touch	0.22 ± 0.22	S*	AccThB	0.25 ± 0.38	S*	AccLB	−0.23 ± 0.25	S*
							AccThB	−0.22 ± 0.34	S*						

Effect sizes are presented as the magnitude of the true difference in means ± 90% confidence interval; only the variables that showed significant differences were included. Positive effect size indicates that the mean values of the variables from group A are bigger than the mean values of the variables from group B; negative effect size indicates that the mean values of the variables from group B are bigger than the mean values of the variables from group A, e.g., group B–group A: group stage–knockout stage. Letters in parentheses denote the magnitude: t, trivial; s, small; m, moderate. Asterisks indicate the likelihood for the magnitude of the true difference in means as follows: * possible; ** likely; *** very likely; **** most likely. CD, central defender; FB, full back; WM, wide midfielder; CM, central midfielder; FW, forward; ShotOT, shot on target; Disp, player is dispossessed on the ball by an opponent-no dribble involved; UnsTouch, Unsuccessful touch; AW, aerial won; YC, yellow card; TT, total tackle; BS, blocked shot; KP, key pass; PA, pass accuracy in %; AccCross, accurate cross pass; LB, long ball; AccLB, accurate long ball; ThB, through ball; AccThB, accurate through ball.

Given the focus on the perspective of situational effects, the variation of technical variables of players from all five playing positions was compared with a focus on each situational variable. Players in the knockout stage showed higher variation in most of the variables related to passing and organising, and lower variation in the other three types of technical variables compared to players in the group stage. The variability of the match performance of full backs, wide midfielders, central midfielders and forwards was greater when playing in away matches than when playing in home matches. However, central defenders showed the opposite trend as the variation of most variables with significant effects (dispossessed, blocked shot, through ball and accurate through ball) was higher in home matches than in away matches. Yellow cards showed obvious differences of variation between players from qualified and non-qualified teams in most of the playing positions (central defender, wide midfielder, central midfielder and forward). In general, central defenders and full backs from qualified teams showed higher variability in the technical performance than their counterparts from non-qualified teams. The variation of variables related to attacking and variables related to passing and organising for all players was higher when playing against qualified opposing teams than playing against non-qualified opposing teams, whereas the variability of defending related variables from all players was higher when playing against non-qualified opposing teams than playing against qualified opposing teams. Moreover, the variation of match performance for wide midfielders, central midfielders and forwards in winning matches was lower than those in draw/defeated matches.

The differences in players’ technical variation were also examined with a focus on the differences between playing positions. The variation of accurate through balls from central defenders showed significant differences in all five competing contexts and the biggest magnitude of difference can be found in the comparison between different match locations (ES ± 90% CI: −0.63 ± 0.45). Similarly, the difference in the variation of accurate through balls (0.82 ± 0.6) from full backs between match locations was higher than in the other four competing situations. The differences of variation in blocked shots and clearance for full backs were all significant with regard to the situational variables of quality of the team, quality of the opponent and match outcome. The variation of wide midfielders’ performance in blocked shots (0.81 ± 0.39) showed very likely moderate differences under different match locations. Wide midfielders’ variation in variables related to goal scoring (shot or shot on target) showed significant differences in all five competing contexts. Central midfielders’ variation only showed a significant difference in key passes (0.21 ± 0.15) between home and away matches, while the other 24 variables showed trivial differences. Central midfielders moreover showed significant differences in through balls in the situational variables of quality of the team, quality of the opponent and match outcome. For forwards, the performance variation in accurate through balls (−0.72 ± 0.38) also showed very likely to moderate differences between winning matches and draw/defeated matches. Except for the situational variable of competition stage, forwards showed significant differences in variables related to goal scoring (shot or shot on target) under the other four situational variables. Performance variation of forwards showed relative larger differences under situational variables of quality of the team, quality of the opponent and match outcome compared to the other two situational variables.

## Discussion

The current study analysed the variability of the technical performance of players based on a large sample (eight seasons from the UCL) and thus in a more comprehensive and detailed manner than previous research (Bush et al., 2015b; Liu et al., 2016). As argued, the examination of both positional and situational effects on the players’ performance variation can provide a better understanding of the interaction effects across playing positions and situational variables.

Technical variation of all playing positions was compared under five competing contexts. To the best of our knowledge, previous research has not examined the differences of a player’s performance variation between the group stage and the knockout stage of the UEFA Champions League so far. Thus, in the current study, we found that for all playing positions, players from the knockout stage showed more variable performance in passing and organising related variables than players from the group stage. Previous studies have identified that players’ performances in variables related to passing and organising were more inconsistent when playing against strong teams than when playing against weak teams (Liu et al., 2016) and that strong teams tend to achieve a high ratio of ball possession in a match (Jones et al., 2004). The strategy and tactics that teams use against strong opponents are highly influenced by the playing styles and tactics of the opponent, while teams will be able to maintain their usual playing style when playing against weaker opponents (Fernandez-Navarro et al., 2018; Memmert et al., 2019). The opponents in the knockout stage are relatively stronger; this could be one potential reason why players from the knockout stage showed more variable performances in passing and organising related variables.

A prior study analysing data of the Spanish First Division Professional Football League (*La Liga*) has reported that the effect of team and opposition strength on the variation of technical variables of players is greater than the effect of match location and match outcome (Liu et al., 2016), which is inconsistent with the results of the current study. This difference may be due to the higher competitiveness of the UEFA Champions League in comparison with the domestic league; teams attempt to perform at the highest level possible in this elimination competition. The match performance of central defenders and full backs from qualified teams varied more than central defenders and full backs from non-qualified teams. This might be due to the multiple tasks for defenders from qualified teams; they need to accomplish defensive duties while also being involved in the attacking process during the match play (Perl and Memmert, 2017). Defenders from non-qualified teams, in contrast, might be mainly concentrated on defending and their match performance is, therefore, more consistent and predictable. Players from all playing positions showed more consistent performance in variables related to attacking and variables related to passing and organising when playing against non-qualified opposing teams than when playing against qualified opposing teams. While players from weaker teams have a limited capacity in attacking and organisation, stronger teams show better tactical discipline and strategical organisation (Castellano et al., 2012). This might be the reason why players from all playing positions tend to achieve a more stable offensive performance when playing against non-qualified opposing teams. In the meantime, players might face greater defensive pressure when playing against qualified opposing teams than playing against non-qualified opposing teams, which would explain that all playing positions showed more stable performance in variables related to defending when playing against qualified opposing teams.

The technical variation of full backs, wide midfielders, central midfielders and forwards was more stable in home matches than in away matches due to the home advantage. Nevertheless, central defenders showed a more variable performance in dispossessed, blocked shots, through balls and accurate through balls in home matches. Probably, this can be explained by the fact that teams are more likely to adopt offensive tactics when playing at home than away (Pollard, 2008; Poulter, 2009), which provides some chances for them to participate in the attacking process. Thus, a more variable performance was shown in these variables in home matches due to the low frequency of occurrence. When taking the effect of match outcome into account, for those variables that showed significant differences, players from five positions showed more consistent performances in winning matches than in draw/defeated matches, which may support the notion that stable technical performance is beneficial to teams to succeed in a match (Gelade, 2017).

The current study also compared the technical variation between each playing position under five competing contexts to identify the effect of situational variables on the match variation of each playing position. We found that the variation of accurate through balls from central defenders and full backs in home/away matches and from forwards in winning/draw/defeated matches all showed very likely moderate differences. These findings may indicate that the effect of match location has a significant impact on the stability of the defenders’ performance in accurate through balls. This issue could be explained by the positive assertive behaviour displayed by the home players (i.e., better defensive actions due to pressing with reduced contact) compared with the negative assertive behaviour of away players (i.e., poor decision making due to the defensive pressure) (Thomas et al., 2006; Lago-Peñas and Lago-Ballesteros, 2011). In addition, increasing the frequency of occurrence of accurate through balls for forwards might contribute to a better match result for teams (Rein et al., 2017). Interestingly, the significant differences in accurate through balls from central defenders can be found in all five competing contexts, which provides further evidence that accurate through ball is the most sensitive variable during match play and can be easily influenced by competing contexts.

There were a higher number of variables that showed that significant differences were observed under situational variables such as quality of the team, quality of the opponent and match outcome compared to the other two situational variables. This result may indicate that these three situational variables have greater impact on the technical variation of players. This impact can be easy to detect in the variables related to defending (blocked shots and clearance) from the full backs, as these two defensive variables showed significant differences under three competing situations. Moreover, the variation of blocked shots from wide midfielders showed a significant difference between home and away matches. The more unstable performance in away matches might be traced back to the fact that they had to make more attempts than usual to block the opponent’s shot in away matches and a small change in the frequency of occurrence caused a large impact on the CV observed (Carling et al., 2016). The variation of variables related to goal scoring for wide midfielders showed significant differences in all five competing contexts, indicating that the match performance of wide midfielders in variables related to goal scoring is highly dependent on competing contexts. This finding can provide a valuable reference for coaches to utilise in the coaching process.

Previous studies have already reported that the technical performance of players was more stable in home matches than in away matches (Poulter, 2009; Almeida et al., 2014), which could be replicated except for central defenders. Probably, central defenders are responsible for the last line of defence that may generate goals against (Di Salvo et al., 2007); the pressure they faced from opponents with different levels and different playing styles may modify their performance and then reduce the stability of match performance for this position. Another interesting finding of this study is that the match location had limited impact on the variability of the central midfielders’ technical performance. The variation of variables related to passing and organising did not show obvious differences as it could be expected between home and away matches except for key pass. Additionally, we observed significant differences in the variation of through balls for central midfielders in competing contexts of quality of the team, quality of the opponent and match outcome. For forwards, the variation of variables related to goal scoring and the variable of key pass both showed significant differences in the competing contexts of match location, quality of the team, quality of the opponent and match outcome. This finding indicates that those forwards being able to seize the goal scoring opportunities and possess qualities in organising the offensive process by proper passes can make a significant contribution to their team (Dellal et al., 2010).

The current study has some limitations that need to be addressed in future studies. Firstly, some of the match statistics used in the current study can be analysed in depth from a defensive or offensive perspective (e.g., Red and Yellow cards) in order to identify their impact on a player’s performances. Secondly, there is limited literature available on players’ technical variation considering both situational and positional effects, which may restrict further interpretation of the findings by comparing our results with previous studies. Lastly, a great number of pairwise comparisons were conducted across playing positions and situational variables; this may probably result in an increase in type I errors from the perspective of statistics. Moreover, some insights for future research should also be noted. Firstly, the analysis of minute-by-minute variation of players’ performance would help to identify the variability and momentum of their behaviours in relation to match status or match period contextual-related variables (Rein and Memmert, 2016). Secondly, the combination of technical, tactical, physical and positional performance would help to have an overall approach of players’ performances during matches. Lastly, statistical models applied to match statistics should be combined from univariate and multivariate approaches avoiding biasing or masking the effects of situational variables on players’ performances.

## Conclusion

This study established performance profiles for players’ technical variation considering five playing positions and five situational variables to examine their interactions on players’ technical variation from a long-term perspective using one of the largest samples published to date (eight seasons). The large dataset makes it possible for us to conduct a comprehensive analysis, such as the difference between the group stage and the knockout stage, for which existing studies have failed to account. Our results showed that the technical match performance of full backs and wide midfielders was more variable between the group stage and the knockout stage. Situational variables of quality of the team, quality of the opponent and match outcome showed similar associations with the variation of the players’ technical performance. Their impacts on the variability of technical performance were greater than the match location. Furthermore, variables related to goal scoring and variables related to passing and organising from all positions showed relatively higher variation than the other two types of variables in five competing contexts.

The established technical variation profiles can be used for pre-match preparation considering the conditions of the next match and for the players’ post-match evaluation to develop position-specific interventions in the coaching process. The profiles can also provide an important tool for talent identification and players’ recruitment. Performance data of a single player can be integrated into the profiles and be compared with the players from the same position under different competing contexts. Furthermore, coaches and performance analysts should pay more attention to those sensitive indicators, such as shot on target and through ball, that have been identified with high match-to-match variations in the current study.

## Data Availability Statement

This study involved the analysis of publicly available data obtained from football statistics website https://www.whoscored.com.

## Author Contributions

QY, M-ÁG, and HL conceptualised the study. M-ÁG and HL contributed to the methodology. QY contributed to the software, data collection, visualisation, and writing the original draft preparation. M-ÁG, HL, BG, FW, and DM reviewed and edited the manuscript. M-ÁG, HL, FW, and DM supervised the study. BG contributed to the funding acquisition. All authors contributed to the article and approved the submitted version.

## Conflict of Interest

The authors declare that the research was conducted in the absence of any commercial or financial relationships that could be construed as a potential conflict of interest.

## Footnotes

1 https://www.whoscored.com
