# Supplementary material for: Situational and Positional Effects on the Technical Variation of Players in the UEFA Champions League
Source: Front Psychol. 2020 Jun 19;11:1201. doi: 10.3389/fpsyg.2020.01201 (PMC7318796; doi:10.3389/fpsyg.2020.01201)
Supplement: Supplementary file 1 [file Table_1.DOCX]

**Supplementary Materials**

Table S1. Descriptive statistics of the variation of central defenders’ performance under five competing situations (arbitrary unit)

| Variable | Central Defender | | | | | | | | | | | | | |
| --- | --- | --- | --- | --- | --- | --- | --- | --- | --- | --- | --- | --- | --- | --- |
|  | Group | Knockout |  | Home | Away |  | Non-qualified | Qualified |  | Non-qualified Opp. | Qualified Opp. |  | Draw/Lose | Win |
| Shot | 1.38±0.66(338) | 1.42±0.59(94) |  | 1.23±0.6(226) | 1.33±0.57(200) |  | 1.37±0.59(205) | 1.39±0.72(178) |  | 1.22±0.59(212) | 1.34±0.62(219) |  | 1.38±0.6(247) | 1.26±0.58(151) |
| ShotOT | 2.02±0.79(207) | 2.01±0.77(57) |  | 1.67±0.64(138) | 1.92±0.69(98) |  | 1.92±0.75(115) | 1.91±0.64(106) |  | 1.8±0.77(121) | 1.73±0.67(114) |  | 1.91±0.73(130) | 1.73±0.68(95) |
| Disp | 1.59±0.88(310) | 1.58±0.9(92) |  | 1.57±0.68(179) | 1.38±0.67(175) |  | 1.54±0.75(188) | 1.6±0.93(151) |  | 1.43±0.76(165) | 1.49±0.73(193) |  | 1.48±0.74(220) | 1.57±0.78(118) |
| UnsTouch | 1.64±0.73(278) | 1.62±0.54(91) |  | 1.54±0.67(176) | 1.47±0.68(173) |  | 1.6±0.73(165) | 1.67±0.73(148) |  | 1.52±0.66(166) | 1.53±0.67(181) |  | 1.54±0.7(203) | 1.63±0.67(116) |
| Fouled | 1.21±0.6(389) | 1.16±0.59(112) |  | 1.21±0.59(256) | 1.15±0.6(251) |  | 1.14±0.63(239) | 1.27±0.58(195) |  | 1.06±0.6(242) | 1.23±0.57(258) |  | 1.21±0.6(305) | 1.15±0.61(163) |
| AW | 0.94±0.48(370) | 0.96±0.46(121) |  | 0.9±0.46(265) | 0.9±0.49(260) |  | 0.93±0.49(239) | 0.91±0.48(183) |  | 0.83±0.51(255) | 0.92±0.52(263) |  | 0.95±0.51(294) | 0.91±0.49(180) |
| Dribble | 1.62±0.89(286) | 1.51±0.71(77) |  | 1.38±0.68(161) | 1.47±0.64(181) |  | 1.51±0.82(169) | 1.63±0.82(144) |  | 1.45±0.76(159) | 1.5±0.74(182) |  | 1.51±0.75(201) | 1.46±0.66(121) |
| Offside | 2.25±0.96(92) | 2.28±0.74(33) |  | 1.85±0.74(47) | 1.94±0.63(43) |  | 1.85±0.72(50) | 2.23±0.79(45) |  | 2.00±0.7(49) | 1.73±0.76(50) |  | 1.92±0.69(61) | 2.14±0.89(34) |
| YC | 1.85±0.69(260) | 1.74±0.78(79) |  | 1.72±0.61(143) | 1.62±0.58(161) |  | 1.72±0.56(158) | 1.94±0.74(130) |  | 1.69±0.69(140) | 1.6±0.54(167) |  | 1.68±0.6(197) | 1.84±0.65(82) |
| TT | 0.74±0.37(421) | 0.75±0.44(132) |  | 0.76±0.41(292) | 0.72±0.44(297) |  | 0.76±0.39(273) | 0.72±0.36(204) |  | 0.69±0.42(273) | 0.73±0.42(307) |  | 0.71±0.4(333) | 0.72±0.42(187) |
| Interception | 0.67±0.36(427) | 0.66±0.39(133) |  | 0.67±0.38(292) | 0.63±0.41(299) |  | 0.67±0.4(275) | 0.66±0.32(207) |  | 0.66±0.41(277) | 0.69±0.43(311) |  | 0.66±0.4(337) | 0.69±0.41(188) |
| Clearance | 0.62±0.37(416) | 0.61±0.38(133) |  | 0.58±0.34(291) | 0.58±0.37(298) |  | 0.57±0.35(269) | 0.66±0.39(202) |  | 0.61±0.4(276) | 0.55±0.34(302) |  | 0.58±0.37(332) | 0.59±0.37(190) |
| BS | 1.31±0.53(334) | 1.21±0.56(100) |  | 1.31±0.52(223) | 1.14±0.55(237) |  | 1.26±0.57(217) | 1.35±0.53(162) |  | 1.23±0.52(211) | 1.2±0.59(243) |  | 1.29±0.57(257) | 1.3±0.56(150) |
| Foul | 1±0.47(416) | 0.95±0.53(125) |  | 0.99±0.55(280) | 0.99±0.51(285) |  | 1.01±0.49(265) | 0.99±0.48(203) |  | 0.98±0.53(262) | 1.00±0.52(298) |  | 1.01±0.52(325) | 0.98±0.56(181) |
| Assist | 2.45±0.94(67) | 2.47±1.02(21) |  | 2.13±0.58(27) | 2.02±0.64(28) |  | 2.11±0.51(25) | 2.32±0.91(43) |  | 2.12±0.65(39) | 2.01±0.64(21) |  | 2.22±0.78(26) | 2.25±0.66(33) |
| Touch | 0.23±0.12(431) | 0.24±0.16(135) |  | 0.24±0.13(297) | 0.23±0.13(305) |  | 0.23±0.12(277) | 0.22±0.11(209) |  | 0.21±0.12(280) | 0.22±0.13(312) |  | 0.23±0.12(340) | 0.22±0.13(192) |
| KP | 1.67±0.8(301) | 1.51±0.61(82) |  | 1.41±0.56(173) | 1.48±0.73(154) |  | 1.61±0.74(173) | 1.58±0.81(150) |  | 1.49±0.7(169) | 1.54±0.74(173) |  | 1.64±0.69(209) | 1.44±0.72(112) |
| PA | 0.09±0.06(431) | 0.1±0.07(135) |  | 0.09±0.06(297) | 0.09±0.08(305) |  | 0.1±0.06(277) | 0.08±0.06(209) |  | 0.08±0.07(280) | 0.09±0.07(312) |  | 0.09±0.07(340) | 0.08±0.05(192) |
| Pass | 0.3±0.16(431) | 0.3±0.2(135) |  | 0.31±0.17(297) | 0.31±0.17(305) |  | 0.31±0.16(277) | 0.29±0.14(209) |  | 0.28±0.16(280) | 0.3±0.17(312) |  | 0.31±0.17(340) | 0.29±0.17(192) |
| Cross | 1.44±1(246) | 1.58±0.83(71) |  | 1.25±0.81(139) | 1.35±0.82(143) |  | 1.26±0.81(147) | 1.47±0.97(118) |  | 1.35±0.86(144) | 1.25±0.82(143) |  | 1.26±0.85(179) | 1.36±0.99(91) |
| AccCross | 1.77±0.97(146) | 2.01±1.14(40) |  | 1.56±0.73(84) | 1.57±0.81(70) |  | 1.58±0.75(82) | 1.82±1.03(75) |  | 1.65±0.85(83) | 1.53±0.78(76) |  | 1.68±0.81(103) | 1.65±0.78(58) |
| LB | 0.54±0.3(430) | 0.52±0.29(135) |  | 0.5±0.31(297) | 0.52±0.33(304) |  | 0.53±0.3(277) | 0.51±0.29(208) |  | 0.45±0.28(280) | 0.54±0.32(311) |  | 0.54±0.31(340) | 0.5±0.29(191) |
| AccLB | 0.69±0.36(423) | 0.72±0.44(135) |  | 0.64±0.37(294) | 0.7±0.42(302) |  | 0.64±0.36(273) | 0.73±0.37(205) |  | 0.6±0.38(277) | 0.67±0.37(304) |  | 0.7±0.36(334) | 0.63±0.39(190) |
| ThB | 2.06±1.01(127) | 2.13±1.2(37) |  | 1.79±0.71(57) | 1.65±0.71(61) |  | 1.86±0.76(63) | 2.09±1.09(70) |  | 1.79±0.93(66) | 1.73±0.71(54) |  | 1.78±0.63(72) | 1.89±0.99(51) |
| AccThB | 2.25±1.02(72) | 2.82±1.24(16) |  | 2.08±0.62(37) | 1.63±0.64(23) |  | 2.08±0.73(35) | 2.33±1.23(40) |  | 1.82±1.06(33) | 2.01±0.77(29) |  | 1.89±0.58(32) | 2.14±1.01(31) |

Note: Values are presented as mean ± SD. Abbreviations: ShotOT=shot on target; Disp=player is dispossessed on the ball by an opponent-no dribble involved; UnsTouch=Unsuccessful touch; AW=aerial won; YC=yellow card; TT=total tackle; BS=blocked shot; KP=key pass; PA=pass accuracy in %; AccCross=accurate cross pass; LB=long ball; AccLB=accurate long ball; ThB=through ball; AccThB=accurate through ball.

Table S2. Descriptive statistics of the variation of full backs’ performance under five competing situations (arbitrary unit)

| Variable | Full Back | | | | | | | | | | | | | |
| --- | --- | --- | --- | --- | --- | --- | --- | --- | --- | --- | --- | --- | --- | --- |
|  | Group | Knockout |  | Home | Away |  | Non-qualified | Qualified |  | Non-qualified Opp. | Qualified Opp. |  | Draw/Lose | Win |
| Shot | 1.46±0.65(325) | 1.4±0.6(80) |  | 1.33±0.64(226) | 1.36±0.58(181) |  | 1.41±0.67(208) | 1.44±0.57(150) |  | 1.34±0.62(196) | 1.37±0.58(219) |  | 1.44±0.63(250) | 1.35±0.56(75) |
| ShotOT | 2.11±0.81(173) | 1.97±0.66(34) |  | 1.75±0.57(111) | 1.84±0.69(88) |  | 1.84±0.61(98) | 2.16±0.82(84) |  | 1.82±0.65(103) | 1.75±0.62(94) |  | 1.86±0.61(106) | 1.8±0.69(4) |
| Disp | 1.37±0.61(333) | 1.29±0.49(86) |  | 1.33±0.54(218) | 1.24±0.58(218) |  | 1.36±0.55(207) | 1.35±0.65(170) |  | 1.3±0.56(197) | 1.26±0.55(218) |  | 1.26±0.56(250) | 1.36±0.61(143) |
| UnsTouch | 1.35±0.59(316) | 1.22±0.67(93) |  | 1.24±0.54(206) | 1.29±0.56(202) |  | 1.26±0.57(205) | 1.37±0.6(152) |  | 1.24±0.54(187) | 1.22±0.51(216) |  | 1.27±0.56(255) | 1.31±0.56(84) |
| Fouled | 1.15±0.52(387) | 1.03±0.55(104) |  | 1.11±0.54(264) | 1.11±0.58(249) |  | 1.13±0.55(253) | 1.13±0.52(182) |  | 1±0.54(237) | 1.14±0.56(261) |  | 1.13±0.56(305) | 1.06±0.51(124) |
| AW | 1.12±0.55(376) | 1.02±0.47(108) |  | 1.09±0.56(264) | 1.07±0.52(243) |  | 1.13±0.57(241) | 1.07±0.53(180) |  | 1.01±0.52(238) | 1.1±0.55(245) |  | 1.09±0.56(287) | 1.02±0.56(177) |
| Dribble | 1.35±0.66(309) | 1.18±0.63(84) |  | 1.24±0.58(211) | 1.27±0.57(192) |  | 1.28±0.62(192) | 1.36±0.62(153) |  | 1.25±0.58(188) | 1.21±0.59(199) |  | 1.26±0.63(240) | 1.28±0.59(168) |
| Offside | 2.42±0.91(100) | 2.31±0.97(29) |  | 1.97±0.71(68) | 1.89±0.75(52) |  | 2.06±0.82(51) | 2.34±0.89(54) |  | 1.95±0.71(62) | 1.82±0.73(52) |  | 2.06±0.73(63) | 2.07±0.9(174) |
| YC | 1.94±0.71(225) | 1.77±0.7(62) |  | 1.78±0.66(114) | 1.69±0.6(140) |  | 1.84±0.6(137) | 1.95±0.8(102) |  | 1.68±0.6(109) | 1.76±0.62(148) |  | 1.84±0.61(174) | 1.78±0.78(68) |
| TT | 0.69±0.33(414) | 0.61±0.37(117) |  | 0.66±0.39(298) | 0.67±0.38(276) |  | 0.69±0.35(270) | 0.68±0.34(197) |  | 0.66±0.43(268) | 0.67±0.38(295) |  | 0.66±0.36(331) | 0.68±0.43(144) |
| Interception | 0.74±0.36(414) | 0.65±0.4(117) |  | 0.73±0.42(295) | 0.71±0.38(275) |  | 0.72±0.35(269) | 0.73±0.38(198) |  | 0.71±0.42(265) | 0.71±0.37(292) |  | 0.71±0.38(330) | 0.74±0.41(135) |
| Clearance | 0.6±0.31(414) | 0.57±0.35(118) |  | 0.65±0.4(299) | 0.57±0.34(277) |  | 0.57±0.3(269) | 0.64±0.33(198) |  | 0.69±0.36(267) | 0.59±0.35(294) |  | 0.59±0.33(331) | 0.68±0.36(189) |
| BS | 1.6±0.67(314) | 1.52±0.71(82) |  | 1.54±0.67(191) | 1.44±0.67(196) |  | 1.48±0.62(199) | 1.72±0.68(138) |  | 1.65±0.72(169) | 1.46±0.61(208) |  | 1.46±0.61(241) | 1.7±0.8(186) |
| Foul | 0.94±0.5(401) | 0.93±0.48(109) |  | 0.91±0.53(283) | 0.92±0.52(260) |  | 0.92±0.51(259) | 0.96±0.5(194) |  | 0.9±0.51(252) | 0.96±0.52(274) |  | 0.92±0.51(314) | 0.94±0.52(190) |
| Assist | 2.39±0.88(108) | 2.33±0.81(25) |  | 1.97±0.63(71) | 1.96±0.58(44) |  | 2.17±0.79(47) | 2.34±0.99(69) |  | 1.97±0.7(66) | 2.05±0.72(48) |  | 2.17±0.78(34) | 1.89±0.9(45) |
| Touch | 0.20±0.11(415) | 0.22±0.12(118) |  | 0.21±0.13(300) | 0.21±0.12(277) |  | 0.21±0.11(270) | 0.22±0.11(198) |  | 0.2±0.12(268) | 0.21±0.11(296) |  | 0.21±0.11(331) | 0.19±0.12(136) |
| KP | 1.31±0.57(331) | 1.39±0.61(83) |  | 1.12±0.53(235) | 1.29±0.56(203) |  | 1.3±0.56(207) | 1.23±0.57(163) |  | 1.17±0.51(207) | 1.27±0.58(219) |  | 1.28±0.58(252) | 1.19±0.57(44) |
| PA | 0.1±0.06(415) | 0.1±0.07(118) |  | 0.1±0.07(300) | 0.1±0.07(277) |  | 0.1±0.06(270) | 0.1±0.06(198) |  | 0.09±0.07(268) | 0.09±0.07(296) |  | 0.1±0.06(331) | 0.1±0.07(191) |
| Pass | 0.28±0.13(415) | 0.28±0.15(118) |  | 0.27±0.16(300) | 0.27±0.15(277) |  | 0.27±0.13(270) | 0.28±0.15(198) |  | 0.26±0.15(268) | 0.26±0.14(296) |  | 0.26±0.13(331) | 0.24±0.15(155) |
| Cross | 0.85±0.55(343) | 0.8±0.58(94) |  | 0.7±0.44(245) | 0.81±0.52(228) |  | 0.82±0.5(220) | 0.81±0.55(170) |  | 0.73±0.45(223) | 0.77±0.48(242) |  | 0.79±0.48(269) | 0.69±0.49(191) |
| AccCross | 1.41±0.61(282) | 1.33±0.57(68) |  | 1.21±0.61(201) | 1.39±0.6(162) |  | 1.41±0.59(175) | 1.34±0.61(139) |  | 1.23±0.63(170) | 1.37±0.62(188) |  | 1.35±0.59(212) | 1.25±0.61(191) |
| LB | 0.46±0.28(414) | 0.54±0.31(118) |  | 0.52±0.32(299) | 0.59±0.33(277) |  | 0.54±0.31(269) | 0.52±0.26(198) |  | 0.53±0.33(266) | 0.54±0.33(295) |  | 0.54±0.32(330) | 0.51±0.3(156) |
| AccLB | 0.74±0.42(410) | 0.77±0.48(115) |  | 0.7±0.41(294) | 0.8±0.44(266) |  | 0.75±0.44(265) | 0.74±0.42(197) |  | 0.71±0.43(262) | 0.77±0.47(287) |  | 0.74±0.45(323) | 0.72±0.44(120) |
| ThB | 2.47±0.9(92) | 2.33±0.97(24) |  | 1.85±0.55(61) | 2.12±0.68(44) |  | 2.16±0.59(48) | 2.59±1.04(47) |  | 1.95±0.79(53) | 1.91±0.46(46) |  | 2.04±0.53(56) | 1.98±0.86(191) |
| AccThB | 2.42±0.7(33) | 2.75±1.27(8) |  | 1.88±0.47(30) | 2.32±0.55(11) |  | 2.06±0.49(16) | 2.49±0.78(18) |  | 2.04±0.72(23) | 2.18±0.62(14) |  | 2.3±0.56(17) | 2.04±0.62(189) |

Note: Values are presented as mean ± SD. Abbreviations: ShotOT=shot on target; Disp=player is dispossessed on the ball by an opponent-no dribble involved; UnsTouch=Unsuccessful touch; AW=aerial won; YC=yellow card; TT=total tackle; BS=blocked shot; KP=key pass; PA=pass accuracy in %; AccCross=accurate cross pass; LB=long ball; AccLB=accurate long ball; ThB=through ball; AccThB=accurate through ball.

Table S3. Descriptive statistics of the variation of wide midfielders’ performance under five competing situations (arbitrary unit)

| Variable | Wide Midfielder | | | | | | | | | | | | | |
| --- | --- | --- | --- | --- | --- | --- | --- | --- | --- | --- | --- | --- | --- | --- |
|  | Group | Knockout |  | Home | Away |  | Non-qualified | Qualified |  | Non-qualified Opp. | Qualified Opp. |  | Draw/Lose | Win |
| Shot | 0.78±0.51(252) | 0.74±0.49(69) |  | 0.74±0.51(132) | 0.83±0.53(120) |  | 0.81±0.51(147) | 0.74±0.53(115) |  | 0.77±0.46(104) | 0.76±0.53(131) |  | 0.79±0.53(170) | 0.68±0.45(77) |
| ShotOT | 1.19±0.56(197) | 1.08±0.57(53) |  | 1.05±0.56(99) | 1.17±0.53(94) |  | 1.26±0.52(113) | 1.07±0.58(93) |  | 1.1±0.54(80) | 1.14±0.52(100) |  | 1.18±0.55(124) | 1.02±0.6(66) |
| Disp | 0.78±0.47(250) | 0.71±0.53(70) |  | 0.75±0.53(129) | 0.72±0.47(124) |  | 0.79±0.48(145) | 0.79±0.48(115) |  | 0.73±0.53(105) | 0.75±0.5(130) |  | 0.78±0.48(167) | 0.69±0.49(76) |
| UnsTouch | 0.87±0.53(226) | 0.84±0.6(63) |  | 0.85±0.51(115) | 0.82±0.53(106) |  | 0.84±0.5(134) | 0.89±0.55(100) |  | 0.87±0.55(97) | 0.83±0.52(119) |  | 0.89±0.51(157) | 0.81±0.57(66) |
| Fouled | 0.87±0.51(259) | 0.81±0.47(74) |  | 0.83±0.51(130) | 0.83±0.52(123) |  | 0.88±0.53(153) | 0.82±0.5(116) |  | 0.77±0.49(103) | 0.88±0.55(134) |  | 0.84±0.54(177) | 0.82±0.55(78) |
| AW | 1.1±0.58(207) | 1.04±0.6(59) |  | 1.07±0.56(100) | 0.99±0.64(96) |  | 1.16±0.54(118) | 1.08±0.63(96) |  | 1.09±0.55(93) | 1.08±0.55(98) |  | 1.07±0.6(134) | 1.01±0.58(69) |
| Dribble | 1.01±0.6(227) | 0.9±0.6(63) |  | 0.88±0.6(112) | 0.96±0.56(107) |  | 0.96±0.6(131) | 1.02±0.64(104) |  | 0.94±0.58(94) | 0.91±0.59(114) |  | 1.01±0.56(150) | 0.94±0.63(74) |
| Offside | 1.33±0.65(121) | 1.4±0.49(39) |  | 1.18±0.54(62) | 1.34±0.55(56) |  | 1.15±0.62(66) | 1.41±0.59(58) |  | 1.11±0.54(57) | 1.35±0.58(55) |  | 1.26±0.61(73) | 1.17±0.63(41) |
| YC | 1.61±0.6(98) | 1.45±0.49(40) |  | 1.36±0.63(33) | 1.51±0.49(42) |  | 1.47±0.5(56) | 1.68±0.6(41) |  | 1.61±0.47(32) | 1.46±0.38(48) |  | 1.43±0.54(60) | 1.6±0.5(23) |
| TT | 0.83±0.48(255) | 0.72±0.5(66) |  | 0.77±0.52(130) | 0.83±0.54(127) |  | 0.82±0.49(151) | 0.81±0.48(113) |  | 0.74±0.48(102) | 0.79±0.5(132) |  | 0.83±0.5(172) | 0.73±0.51(77) |
| Interception | 0.92±0.54(248) | 0.87±0.51(71) |  | 0.93±0.53(125) | 0.81±0.56(116) |  | 0.89±0.54(150) | 0.95±0.55(105) |  | 0.85±0.58(100) | 0.96±0.55(134) |  | 0.91±0.53(163) | 0.9±0.57(76) |
| Clearance | 1.05±0.65(222) | 1.13±0.56(59) |  | 1.01±0.64(105) | 0.96±0.63(107) |  | 1±0.63(125) | 1.07±0.58(100) |  | 1.11±0.56(94) | 0.99±0.67(108) |  | 1.01±0.6(148) | 0.97±0.63(66) |
| BS | 1.61±0.64(85) | 1.45±0.61(28) |  | 1.33±0.43(37) | 1.5±0.61(40) |  | 1.55±0.52(45) | 1.57±0.62(38) |  | 1.32±0.7(27) | 1.42±0.44(42) |  | 1.54±0.57(52) | 1.52±0.45(29) |
| Foul | 0.91±0.52(255) | 0.77±0.5(74) |  | 0.87±0.58(128) | 0.89±0.54(121) |  | 0.89±0.52(149) | 0.92±0.53(114) |  | 0.86±0.53(101) | 0.93±0.5(129) |  | 0.92±0.53(172) | 0.81±0.53(73) |
| Assist | 1.75±0.55(76) | 1.73±0.57(23) |  | 1.5±0.46(36) | 1.62±0.36(33) |  | 1.72±0.53(34) | 1.72±0.52(46) |  | 1.56±0.61(36) | 1.67±0.46(28) |  | 1.68±0.53(26) | 1.42±0.51(39) |
| Touch | 0.19±0.1(268) | 0.24±0.21(75) |  | 0.18±0.11(138) | 0.18±0.12(133) |  | 0.18±0.1(159) | 0.19±0.11(119) |  | 0.17±0.12(110) | 0.18±0.11(141) |  | 0.18±0.11(182) | 0.18±0.11(83) |
| KP | 0.96±0.52(235) | 0.9±0.48(66) |  | 0.84±0.55(121) | 0.97±0.53(113) |  | 0.91±0.54(139) | 0.97±0.49(106) |  | 0.92±0.5(100) | 0.94±0.54(124) |  | 0.96±0.56(161) | 0.81±0.5(70) |
| PA | 0.09±0.07(268) | 0.11±0.09(75) |  | 0.09±0.08(138) | 0.09±0.09(133) |  | 0.09±0.07(159) | 0.09±0.07(119) |  | 0.08±0.06(110) | 0.1±0.08(141) |  | 0.1±0.08(182) | 0.09±0.07(83) |
| Pass | 0.23±0.13(268) | 0.29±0.22(75) |  | 0.22±0.14(138) | 0.23±0.15(133) |  | 0.22±0.12(159) | 0.24±0.14(119) |  | 0.22±0.15(110) | 0.21±0.13(141) |  | 0.22±0.13(182) | 0.24±0.15(83) |
| Cross | 0.83±0.56(227) | 0.82±0.49(59) |  | 0.73±0.49(118) | 0.86±0.52(112) |  | 0.75±0.53(133) | 0.85±0.57(100) |  | 0.77±0.54(92) | 0.77±0.51(120) |  | 0.75±0.52(152) | 0.85±0.54(71) |
| AccCross | 1.22±0.58(149) | 1.23±0.59(37) |  | 1.15±0.52(80) | 1.25±0.55(69) |  | 1.16±0.57(86) | 1.22±0.59(69) |  | 1.13±0.6(67) | 1.21±0.48(76) |  | 1.19±0.55(93) | 1.16±0.56(48) |
| LB | 0.73±0.48(251) | 0.72±0.51(71) |  | 0.67±0.46(127) | 0.69±0.45(120) |  | 0.73±0.49(147) | 0.72±0.48(113) |  | 0.75±0.53(100) | 0.71±0.44(132) |  | 0.73±0.48(169) | 0.63±0.46(78) |
| AccLB | 0.89±0.57(235) | 0.85±0.51(66) |  | 0.84±0.54(117) | 0.85±0.53(111) |  | 0.91±0.6(137) | 0.86±0.53(105) |  | 0.85±0.53(90) | 0.88±0.57(123) |  | 0.87±0.59(153) | 0.77±0.54(74) |
| ThB | 1.51±0.62(117) | 1.45±0.41(36) |  | 1.35±0.56(53) | 1.37±0.59(52) |  | 1.54±0.57(53) | 1.41±0.56(65) |  | 1.46±0.59(58) | 1.33±0.52(55) |  | 1.47±0.58(64) | 1.28±0.48(44) |
| AccThB | 1.85±0.67(66) | 1.68±0.4(21) |  | 1.36±0.6(32) | 1.55±0.63(28) |  | 1.84±0.43(23) | 1.74±0.57(41) |  | 1.6±0.67(36) | 1.5±0.57(28) |  | 1.7±0.59(34) | 1.55±0.63(29) |

Note: Values are presented as mean ± SD. Abbreviations: ShotOT=shot on target; Disp=player is dispossessed on the ball by an opponent-no dribble involved; UnsTouch=Unsuccessful touch; AW=aerial won; YC=yellow card; TT=total tackle; BS=blocked shot; KP=key pass; PA=pass accuracy in %; AccCross=accurate cross pass; LB=long ball; AccLB=accurate long ball; ThB=through ball; AccThB=accurate through ball.

Table S4. Descriptive statistics of the variation of central midfielders’ performance under five competing situations (arbitrary unit)

| Variable | Central Midfielder | | | | | | | | | | | | | |
| --- | --- | --- | --- | --- | --- | --- | --- | --- | --- | --- | --- | --- | --- | --- |
|  | Group | Knockout |  | Home | Away |  | Non-qualified | Qualified |  | Non-qualified Opp. | Qualified Opp. |  | Draw/Lose | Win |
| Shot | 1.06±0.57(474) | 1.02±0.53(135) |  | 0.99±0.57(308) | 1.07±0.55(290) |  | 1.04±0.57(279) | 1.07±0.59(245) |  | 0.92±0.56(275) | 1.06±0.55(297) |  | 1.1±0.55(336) | 0.94±0.56(215) |
| ShotOT | 1.54±0.74(334) | 1.43±0.61(102) |  | 1.37±0.6(202) | 1.52±0.59(174) |  | 1.51±0.65(186) | 1.51±0.77(174) |  | 1.28±0.63(186) | 1.55±0.59(186) |  | 1.56±0.63(208) | 1.42±0.66(158) |
| Disp | 1.03±0.59(460) | 0.97±0.54(130) |  | 1.01±0.54(297) | 0.97±0.59(288) |  | 1.02±0.52(263) | 1.04±0.65(240) |  | 0.96±0.58(273) | 1±0.53(288) |  | 0.99±0.57(320) | 1.01±0.59(209) |
| UnsTouch | 1.15±0.6(411) | 1.09±0.55(125) |  | 1.12±0.59(270) | 1.1±0.6(265) |  | 1.07±0.59(232) | 1.19±0.6(220) |  | 1.08±0.58(255) | 1.11±0.57(252) |  | 1.13±0.59(287) | 1.15±0.59(197) |
| Fouled | 0.95±0.49(480) | 0.89±0.51(147) |  | 0.94±0.53(323) | 0.91±0.53(307) |  | 0.97±0.52(278) | 0.89±0.47(252) |  | 0.9±0.52(299) | 0.94±0.52(315) |  | 0.96±0.51(341) | 0.88±0.53(239) |
| AW | 1.2±0.59(437) | 1.08±0.59(135) |  | 1.07±0.58(283) | 1.12±0.53(268) |  | 1.2±0.56(251) | 1.15±0.58(227) |  | 1.04±0.53(263) | 1.18±0.56(278) |  | 1.19±0.57(306) | 1.01±0.53(220) |
| Dribble | 1.25±0.6(397) | 1.15±0.63(126) |  | 1.16±0.53(237) | 1.18±0.58(248) |  | 1.22±0.57(231) | 1.24±0.62(207) |  | 1.13±0.61(230) | 1.21±0.53(246) |  | 1.2±0.57(276) | 1.11±0.6(190) |
| Offside | 2.01±0.94(158) | 2.05±0.85(34) |  | 1.65±0.69(89) | 1.84±0.68(63) |  | 1.78±0.72(70) | 1.96±0.91(93) |  | 1.7±0.67(81) | 1.78±0.64(68) |  | 1.74±0.73(88) | 1.84±0.69(63) |
| YC | 1.82±0.67(282) | 1.63±0.66(92) |  | 1.63±0.5(139) | 1.54±0.63(180) |  | 1.68±0.6(171) | 1.87±0.68(134) |  | 1.69±0.65(129) | 1.58±0.56(175) |  | 1.61±0.65(202) | 1.7±0.64(97) |
| TT | 0.71±0.4(509) | 0.7±0.47(156) |  | 0.69±0.45(346) | 0.69±0.46(339) |  | 0.71±0.43(299) | 0.68±0.41(263) |  | 0.69±0.43(316) | 0.68±0.4(337) |  | 0.73±0.42(368) | 0.65±0.44(254) |
| Interception | 0.79±0.42(494) | 0.76±0.48(150) |  | 0.74±0.48(338) | 0.78±0.47(335) |  | 0.82±0.43(288) | 0.75±0.42(260) |  | 0.74±0.47(313) | 0.77±0.44(330) |  | 0.81±0.47(357) | 0.74±0.47(255) |
| Clearance | 0.95±0.54(459) | 0.95±0.55(141) |  | 0.94±0.55(311) | 0.87±0.53(311) |  | 0.91±0.54(271) | 0.97±0.54(232) |  | 0.94±0.56(289) | 0.85±0.52(311) |  | 0.9±0.53(328) | 0.9±0.58(230) |
| BS | 1.61±0.7(316) | 1.55±0.71(90) |  | 1.54±0.59(176) | 1.39±0.59(213) |  | 1.48±0.57(179) | 1.71±0.75(161) |  | 1.53±0.63(173) | 1.43±0.66(203) |  | 1.47±0.67(214) | 1.48±0.62(141) |
| Foul | 0.89±0.46(495) | 0.79±0.52(150) |  | 0.87±0.49(331) | 0.81±0.5(324) |  | 0.88±0.48(294) | 0.9±0.45(256) |  | 0.84±0.49(306) | 0.86±0.5(324) |  | 0.86±0.49(357) | 0.85±0.49(244) |
| Assist | 2.12±0.72(148) | 2.06±0.72(56) |  | 1.86±0.62(82) | 1.85±0.57(70) |  | 2.11±0.61(61) | 2±0.68(97) |  | 1.79±0.6(97) | 1.91±0.56(55) |  | 1.98±0.72(52) | 1.73±0.6(87) |
| Touch | 0.21±0.11(515) | 0.21±0.13(158) |  | 0.21±0.12(351) | 0.21±0.12(343) |  | 0.21±0.11(304) | 0.22±0.12(265) |  | 0.2±0.13(324) | 0.2±0.12(344) |  | 0.21±0.13(375) | 0.21±0.12(258) |
| KP | 1.1±0.57(431) | 1.08±0.6(126) |  | 1±0.54(283) | 1.1±0.56(252) |  | 1.12±0.56(253) | 1.06±0.58(224) |  | 1±0.54(258) | 1.09±0.54(264) |  | 1.09±0.55(300) | 1.02±0.56(201) |
| PA | 0.08±0.05(515) | 0.08±0.06(158) |  | 0.08±0.06(351) | 0.08±0.06(343) |  | 0.08±0.05(304) | 0.07±0.04(265) |  | 0.08±0.05(324) | 0.08±0.05(344) |  | 0.08±0.05(375) | 0.08±0.06(258) |
| Pass | 0.27±0.14(515) | 0.27±0.16(158) |  | 0.26±0.15(351) | 0.26±0.16(343) |  | 0.26±0.14(304) | 0.27±0.15(265) |  | 0.25±0.16(324) | 0.25±0.14(344) |  | 0.25±0.15(375) | 0.27±0.16(258) |
| Cross | 1.19±0.73(371) | 1.11±0.69(109) |  | 1.06±0.62(244) | 1.13±0.64(210) |  | 1.12±0.69(207) | 1.19±0.69(198) |  | 1.09±0.63(230) | 1.07±0.67(204) |  | 1.1±0.65(253) | 1.15±0.63(178) |
| AccCross | 1.53±0.8(242) | 1.49±0.73(65) |  | 1.38±0.69(150) | 1.41±0.65(127) |  | 1.45±0.71(134) | 1.53±0.75(129) |  | 1.34±0.71(136) | 1.34±0.7(125) |  | 1.41±0.7(167) | 1.39±0.65(106) |
| LB | 0.55±0.31(510) | 0.55±0.34(157) |  | 0.51±0.33(347) | 0.54±0.36(341) |  | 0.53±0.32(301) | 0.56±0.3(263) |  | 0.53±0.35(323) | 0.53±0.31(341) |  | 0.53±0.34(371) | 0.53±0.33(256) |
| AccLB | 0.7±0.4(507) | 0.68±0.4(153) |  | 0.62±0.39(341) | 0.67±0.44(336) |  | 0.67±0.4(300) | 0.7±0.38(261) |  | 0.66±0.41(315) | 0.65±0.41(339) |  | 0.67±0.42(367) | 0.65±0.43(256) |
| ThB | 1.63±0.75(259) | 1.62±0.64(86) |  | 1.4±0.6(147) | 1.52±0.6(144) |  | 1.66±0.66(126) | 1.54±0.72(146) |  | 1.38±0.62(153) | 1.55±0.58(131) |  | 1.6±0.64(161) | 1.4±0.66(113) |
| AccThB | 2.04±0.72(153) | 2±0.98(54) |  | 1.72±0.6(86) | 1.82±0.59(78) |  | 1.93±0.61(66) | 1.92±0.63(90) |  | 1.69±0.58(93) | 1.93±0.54(72) |  | 1.87±0.55(80) | 1.73±0.68(72) |

Note: Values are presented as mean ± SD. Abbreviations: ShotOT=shot on target; Disp=player is dispossessed on the ball by an opponent-no dribble involved; UnsTouch=Unsuccessful touch; AW=aerial won; YC=yellow card; TT=total tackle; BS=blocked shot; KP=key pass; PA=pass accuracy in %; AccCross=accurate cross pass; LB=long ball; AccLB=accurate long ball; ThB=through ball; AccThB=accurate through ball.

Table S5. Descriptive statistics of the variation of forwards’ performance under five competing situations (arbitrary unit)

| Variable | Forward | | | | | | | | | | | | | |
| --- | --- | --- | --- | --- | --- | --- | --- | --- | --- | --- | --- | --- | --- | --- |
|  | Group | Knockout |  | Home | Away |  | Non-qualified | Qualified |  | Non-qualified Opp. | Qualified Opp. |  | Draw/Lose | Win |
| Shot | 0.6±0.35(203) | 0.67±0.42(52) |  | 0.53±0.37(125) | 0.62±0.41(118) |  | 0.62±0.39(113) | 0.57±0.31(115) |  | 0.49±0.33(112) | 0.67±0.41(113) |  | 0.63±0.4(151) | 0.54±0.35(79) |
| ShotOT | 1±0.52(187) | 1.07±0.49(49) |  | 0.87±0.54(115) | 1.01±0.48(104) |  | 1.04±0.54(104) | 0.92±0.51(105) |  | 0.86±0.54(104) | 1±0.53(101) |  | 1.03±0.55(129) | 0.81±0.47(76) |
| Disp | 0.79±0.42(201) | 0.76±0.41(52) |  | 0.7±0.43(119) | 0.76±0.45(118) |  | 0.79±0.44(111) | 0.76±0.46(113) |  | 0.74±0.45(107) | 0.76±0.45(113) |  | 0.74±0.43(150) | 0.77±0.46(75) |
| UnsTouch | 0.73±0.43(187) | 0.72±0.41(46) |  | 0.73±0.48(112) | 0.79±0.45(112) |  | 0.68±0.43(100) | 0.76±0.48(109) |  | 0.8±0.5(102) | 0.75±0.43(107) |  | 0.72±0.46(143) | 0.79±0.48(70) |
| Fouled | 0.82±0.46(200) | 0.83±0.46(51) |  | 0.78±0.5(121) | 0.79±0.53(115) |  | 0.8±0.52(113) | 0.79±0.4(111) |  | 0.82±0.47(112) | 0.77±0.51(109) |  | 0.76±0.51(150) | 0.79±0.46(78) |
| AW | 1.05±0.67(171) | 1.09±0.58(37) |  | 1.04±0.63(101) | 1±0.61(93) |  | 1±0.61(92) | 1.07±0.66(96) |  | 1.03±0.65(90) | 0.9±0.61(91) |  | 0.98±0.62(122) | 1±0.56(61) |
| Dribble | 1.03±0.57(183) | 1.06±0.54(46) |  | 0.99±0.56(106) | 0.93±0.57(100) |  | 1.1±0.62(95) | 0.93±0.53(107) |  | 0.97±0.53(100) | 1.1±0.61(100) |  | 1.07±0.59(136) | 0.95±0.52(67) |
| Offside | 1.26±0.55(161) | 1.34±0.61(42) |  | 1.12±0.56(100) | 1.21±0.52(88) |  | 1.33±0.56(86) | 1.15±0.55(91) |  | 1.17±0.58(90) | 1.21±0.51(86) |  | 1.26±0.55(112) | 1.05±0.57(67) |
| YC | 1.84±0.66(99) | 1.83±0.84(29) |  | 1.82±0.65(55) | 1.74±0.79(49) |  | 1.64±0.5(48) | 1.87±0.7(62) |  | 1.75±0.53(49) | 1.62±0.43(50) |  | 1.96±0.65(70) | 1.6±0.84(33) |
| TT | 1.03±0.55(192) | 1.07±0.43(46) |  | 0.99±0.56(110) | 1.06±0.61(110) |  | 1±0.57(98) | 1.02±0.56(110) |  | 1.04±0.58(100) | 1.02±0.59(102) |  | 1.02±0.58(133) | 1.03±0.53(73) |
| Interception | 1.2±0.69(173) | 1.31±0.51(44) |  | 1.2±0.62(98) | 1.2±0.64(95) |  | 1.16±0.62(92) | 1.27±0.7(98) |  | 1.21±0.66(90) | 1.19±0.6(85) |  | 1.26±0.61(126) | 1.13±0.67(60) |
| Clearance | 1.42±0.67(144) | 1.27±0.58(31) |  | 1.32±0.54(73) | 1.23±0.64(80) |  | 1.35±0.58(79) | 1.4±0.71(79) |  | 1.35±0.63(72) | 1.25±0.53(73) |  | 1.34±0.59(97) | 1.36±0.67(54) |
| BS | 2.53±0.88(57) | 2.15±1.23(19) |  | 1.74±0.44(27) | 1.9±0.7(30) |  | 1.91±0.51(29) | 2.15±1.08(28) |  | 1.85±0.62(24) | 1.89±0.62(26) |  | 1.83±0.57(30) | 1.72±0.32(14) |
| Foul | 0.94±0.47(195) | 0.84±0.48(48) |  | 0.84±0.52(114) | 0.92±0.52(107) |  | 0.89±0.52(108) | 0.93±0.47(108) |  | 0.92±0.5(107) | 0.95±0.5(106) |  | 0.96±0.51(145) | 0.91±0.5(74) |
| Assist | 1.79±0.72(89) | 2.01±0.85(24) |  | 1.64±0.5(54) | 1.73±0.59(42) |  | 1.67±0.75(33) | 1.72±0.62(59) |  | 1.64±0.63(58) | 1.72±0.4(33) |  | 1.8±0.59(42) | 1.5±0.64(45) |
| Touch | 0.2±0.1(206) | 0.19±0.11(52) |  | 0.19±0.11(125) | 0.2±0.13(120) |  | 0.18±0.1(114) | 0.21±0.11(116) |  | 0.2±0.12(112) | 0.2±0.12(113) |  | 0.19±0.11(153) | 0.2±0.12(79) |
| KP | 0.86±0.48(195) | 0.91±0.47(50) |  | 0.7±0.48(121) | 0.9±0.53(108) |  | 0.88±0.56(108) | 0.78±0.42(110) |  | 0.77±0.42(108) | 0.87±0.56(102) |  | 0.85±0.52(142) | 0.67±0.44(75) |
| PA | 0.11±0.08(206) | 0.1±0.06(52) |  | 0.1±0.08(125) | 0.1±0.08(120) |  | 0.11±0.08(114) | 0.11±0.09(116) |  | 0.1±0.07(112) | 0.1±0.08(113) |  | 0.1±0.07(153) | 0.11±0.09(79) |
| Pass | 0.24±0.12(206) | 0.22±0.13(52) |  | 0.23±0.13(125) | 0.25±0.16(120) |  | 0.22±0.11(114) | 0.26±0.13(116) |  | 0.24±0.14(112) | 0.24±0.14(113) |  | 0.23±0.13(153) | 0.24±0.14(79) |
| Cross | 0.9±0.55(179) | 0.96±0.52(51) |  | 0.85±0.58(111) | 0.92±0.56(105) |  | 0.89±0.59(99) | 0.89±0.55(100) |  | 0.87±0.54(97) | 0.96±0.58(97) |  | 0.92±0.56(129) | 0.88±0.53(73) |
| AccCross | 1.45±0.69(127) | 1.51±0.56(37) |  | 1.3±0.61(82) | 1.45±0.63(60) |  | 1.36±0.57(63) | 1.41±0.69(71) |  | 1.29±0.6(69) | 1.32±0.65(54) |  | 1.33±0.64(79) | 1.33±0.74(49) |
| LB | 0.94±0.53(194) | 0.91±0.53(47) |  | 0.93±0.52(115) | 0.92±0.56(110) |  | 0.93±0.57(103) | 0.94±0.5(112) |  | 0.9±0.48(108) | 1±0.57(101) |  | 1±0.59(142) | 0.84±0.51(78) |
| AccLB | 1.06±0.57(178) | 1.08±0.62(43) |  | 1.03±0.52(105) | 1.05±0.55(98) |  | 1.04±0.62(91) | 1.06±0.52(104) |  | 1.03±0.5(100) | 1.18±0.62(92) |  | 1.12±0.58(125) | 0.96±0.51(71) |
| ThB | 1.42±0.68(127) | 1.41±0.73(37) |  | 1.29±0.66(70) | 1.38±0.6(69) |  | 1.47±0.66(60) | 1.4±0.66(80) |  | 1.19±0.55(67) | 1.34±0.6(54) |  | 1.41±0.64(76) | 1.25±0.63(57) |
| AccThB | 1.81±0.71(90) | 1.94±0.66(27) |  | 1.59±0.63(49) | 1.54±0.68(43) |  | 1.76±0.42(38) | 1.65±0.76(53) |  | 1.47±0.71(46) | 1.7±0.62(34) |  | 1.69±0.59(48) | 1.39±0.68(36) |

Note: Values are presented as mean ± SD. Abbreviations: ShotOT=shot on target; Disp=player is dispossessed on the ball by an opponent-no dribble involved; UnsTouch=Unsuccessful touch; AW=aerial won; YC=yellow card; TT=total tackle; BS=blocked shot; KP=key pass; PA=pass accuracy in %; AccCross=accurate cross pass; LB=long ball; AccLB=accurate long ball; ThB=through ball; AccThB=accurate through ball.
